# The Relationship Between Prenatal Alcohol Exposure and Infant/Child–Caregiver Attachment: A Scoping Review

**DOI:** 10.3390/children12091133

**Published:** 2025-08-27

**Authors:** David J. Gilbert, Alan D. Price, Penny A. Cook, Raja A. S. Mukherjee

**Affiliations:** 1 School of Health and Society, University of Salford, Manchester M5 4WT, UK; a.d.price3@salford.ac.uk (A.D.P.); p.a.cook@salford.ac.uk (P.A.C.); raja.mukherjee@sabp.nhs.uk (R.A.S.M.); 2 FASD Specialist Behaviour Clinic, Surrey, and Borders Partnership, NHS Foundation Trust Redhill, Surrey RH1 1TA, UK

**Keywords:** attachment styles, attachment security, prenatal alcohol exposure, fetal alcohol spectrum disorder, scoping review

## Abstract

**Introduction:** Secure infant/child–caregiver attachment is crucial for the development of social and emotional functioning and can affect long-term outcomes, such as adult relationships, but it may also be influenced by prenatal and early childhood risk factors. Children with a history of prenatal alcohol exposure (PAE) have a complex spectrum of strengths and difficulties and often have the additional risk of early life adversity. There is some evidence that children with PAE are at increased risk of insecure attachment, but it is unclear whether this is consistent or why it is the case. No published review has focused on the relationship between PAE and attachment. **Methods:** A systematic search of seven academic databases using the PRISMA Extension for Scoping Reviews (PRISMA-ScR) guidelines was undertaken by two reviewers to identify primary studies that have focused on the relationship between PAE and attachment. Quality assessments were undertaken using the Quality of Observational Cohort and Cross-Sectional Studies tool, and the report was written following the PRISMA-ScR checklist. **Results:** A total of 4199 records were returned from the database searches. A total of 11 studies (eight peer-reviewed papers and three dissertations), published between 1987 and 2021, met the criteria. Five studies showed that PAE was related to insecure or disorganised attachment, two of which showed that infant irritability and caregiver–infant interaction mediated this relationship. The other six studies found no significant relationship between PAE and attachment. **Conclusions:** This scoping review demonstrates that there is a dearth of published research on this topic, and none that takes advantage of more recent understanding of the relationship between adverse childhood experiences and neurodevelopmental disorders. There is some evidence that PAE may impact the attachment relationship via caregiver–infant interaction and infant irritability, but further studies, including those that assess the additional impact of early life adversity, are needed.

## 1. Introduction

Prenatal alcohol exposure (PAE) can cause teratogenic changes to a developing foetus [1,2]. These changes can lead to fetal alcohol spectrum disorder (FASD), a common neurodevelopmental condition [3]. FASD is characterised by a range of difficulties with motor skills, cognition, language, academic achievement, memory, attention, executive function, affect regulation, adaptive behaviour, social communication, and sometimes limited growth and craniofacial dysmorphia [4]. The extent of impairment from PAE depends on the frequency, dosage, and timing of alcohol use in pregnancy [5,6], as well as genetic and other factors, e.g., smoking and diet [7]. Children with FASD are at increased risk of adverse childhood experiences (ACEs) [8,9,10,11] and are over-represented in care and justice systems [12,13]. They may also experience multiple placements [14], all of which are potentially disruptive to the healthy development of an attachment relationship. The reason for overrepresentation in the care system is partly due to parental substance abuse and neglect [15]. The rates found within the care system were 32 and 40 times higher than the rates in the general population in, for example, the USA and Chile [12]. For the justice system, previous research shows rates of 46% and 36% within the justice systems of Canada and Australia respectively [16,17]. Vulnerabilities such as impulsivity, suggestibility, confabulation, and executive and adaptive function impairments may be responsible for this high rate of justice system encounters [18,19,20,21]. Despite the above-mentioned vulnerabilities, individuals with FASD also possess many strengths, e.g., artistic, music and computer skills [8]. Additionally, appropriate intervention and support are evidenced to improve outcomes. For example, a systematic review found improved outcomes in language, literacy, executive function, and emotional problem-solving skills via interventions [22].

Attachment theory posits that the infant–caregiver attachment relationship is fundamental to healthy psychosocial development [23]. It is likely an evolutionary adaptation that compels children to seek resources and support from their primary caregiver, and in some cultures, from closely related family members [24]. Children use their caregiver(s) as a secure base from which to explore their environment, returning to them for comfort or safety [25]. Individual differences in attachment styles are typically categorised into four distinct styles. First identified by [26] were the three styles of secure, insecure–avoidant, and insecure–ambivalent/resistant attachment. A secure attachment style (which is usually the most common, at least in Western countries) is seen as resulting from responsive caregiving, and securely attached children are more easily soothed than insecurely attached children [26]. An insecure–avoidant attachment style is characterised by less responsive caregiving and a child who is less likely to seek comfort from the caregiver, whilst a child with an insecure–ambivalent/resistant attachment style may show some level of conflict between seeking and not seeking support [26]. Later, Main and Solomon [27] added a fourth category of disorganised attachment. This attachment style is associated with parenting that is chaotic or unpredictable, and children with this attachment style may appear fearful or avoidant of their caregivers. Children who have been maltreated by their caregivers are likely to have insecure or disorganised attachment styles [28]. Different tools are available for measuring attachment; interviews and questionnaires are available for older children, adolescents, and adults [29,30], whilst the strange situation procedure (SSP; [26]) is commonly used for assessing attachment styles in younger children. This experimental procedure (for the SSP) involves observation of an infant–caregiver dyad along with another adult who is not known to the infant. During a series of separations and reunions, the behaviour of the infant is used to determine which style of attachment they have to their caregiver. The choice of appropriate tools should consider a variety of factors, including but not limited to cultural background, mental health status, level of command of English, and the age of the participant [31,32].

Attachment security in childhood is associated with healthier development and tends to predict security in adult relationships [33,34]. Attachment insecurity in childhood has been linked with more negative outcomes in social and cognitive functioning, physical health, and mental health [35]. The development of a healthy attachment relationship depends on the extent to which both the infant and their primary caregiver are capable of social communication and interaction, amongst other factors [36,37]. For example, emotional availability and the internal working model (IWM) of caregivers are suggested to impact interaction between children and their caregivers [38,39]. IWM defines the frameworks that the caregivers themselves have developed early in their lives, which then shapes the way they see others and establish relationships [38]. The concept of IWM explains the representations (mentally) that individuals possess of themselves and others due to early attachment experiences, thereby influencing their behaviours in future relationships. While emotional availability of the caregiver impacts the extent to which the child may relate with the caregiver, IWM could impact the way the caregiver establishes interaction with the child.

Studies of children with other neurodevelopmental disorders, such as Autism Spectrum Disorder (ASD), have shown that, while secure attachment styles are fairly common, they are less prevalent in comparison to typically developing children [40]. This may be partly due to difficulties with social interaction in children with neurodevelopmental disorders [41]. Furthermore, caregivers who consume higher amounts of alcohol are also less likely to form secure attachment relationships with their infants [42]. Hypothetically, in the case of a dyad where PAE has led to congenital differences in social communication or social interaction in the infant, and where the caregiver is a regular consumer of alcohol, there may be a further increased risk of insecure or disorganised attachment since both the infant and caregiver are affected by those individual risk factors. This is especially important, as there is evidence of increased maltreatment, including but not limited to abuse, trauma, and neglect [9,43]. Evidence shows that young people with FASD had 3.7 points higher levels of adverse experiences compared to non-FASD controls [44]. Rockhold et al. [43] found that 88% of children with FASD experienced three or more categories of trauma. These high rates of maltreatment and adverse experiences may suggest the development of unhealthy attachment styles.

Given the elevated risk associated with higher levels of ACEs in the population affected by PAE [9,10,11], there is a need to assess attachment security in caregiver–infant dyads where FASD is either confirmed or suspected [9,11]. Studies on caregiver–child interaction in those with PAE/FASD have shown that PAE is associated with more negative interaction styles on the part of caregivers [45], as well as difficulties with affect regulation in infants [46], both of which may increase the risk of attachment difficulties. While these studies provide useful insight into the impact of combined substance use and interaction style on child development, there is a need to specifically understand the impact of PAE on attachment styles. There are no systematic reviews of PAE and infant/child–caregiver attachment in the published literature, which limits the extent of understanding the nature of evidence in this area. Interventions are available to address insecure attachment in typically developing individuals, such as, for example, the Attachment and Biobehavioural Catch-Up intervention, Child–Parent Psychotherapy, and Promoting First Relationships (PFR) [47,48,49,50]. Similar and modified interventions/support may also be useful to improve the outcomes for individuals with PAE/FASD. Recognition and identification of the impact of PAE/FASD on attachment is a vital first step in the development of such interventions. The aim of this scoping review is to identify all existing primary studies that have studied the relationship between PAE/FASD and infant/child–caregiver attachment. The research question for the current review was: What is the available evidence regarding the relationship between PAE and infant/child–caregiver attachment? Employing a scoping review approach provides an opportunity to systematically identify available research in this area, in order to analyse gaps within the field and understand the nature of the available evidence [51,52].

## 2. Methods

### 2.1. Eligibility Criteria

This study is a narrative scoping review, hence, did not fit the criteria for registration on PROSPERO. The PRISMA Extension for Scoping Reviews (PRISMA-ScR) guideline was followed in this scoping review as published by [53].

Studies were considered eligible if they were published in English and reported primary research that assessed the relationship between PAE/FASD and infant/child–caregiver attachment, including observational and experimental studies using humans or animals. Theses/dissertations that reported primary research data were included. No limits were placed on dates during the search to ensure that all publications relevant to the topic of the current review are not missed.

#### Exclusion Criteria

The following were used as exclusion criteria: studies that do not separate the effect of alcohol from the effect of other drugs, intervention studies, literature reviews, trials, protocols, studies using tissue samples, and editorials/opinion pieces.

### 2.2. Information Sources

Seven databases (MEDLINE, CINAHL, PsycINFO, PubMed, ERIC, Web of Science (all databases), and Child Development and Adolescent Studies) were searched by the first and second authors. Three rounds of searches were undertaken independently by the first and second authors of the current article: the first set of searches was conducted on 20 May 2021, while the second set of systematic searches was carried out on 17 June 2021, and an updated search was conducted prior to submission on 1 September 2023.

### 2.3. Search and Study Selection

The following search terms were used to search the abstracts (topic in Web of Science) of records across the seven databases:

 

FASD OR “fetal alcohol” OR “foetal alcohol” OR “prenatal alcohol” OR “pre-natal alcohol” OR “pre-natal alcohol” OR “prenatal ethanol” OR “pre-natal ethanol” OR “pre-natal ethanol” OR “alcohol in pregnancy” OR “alcohol in utero” AND attachment OR disinhibited OR indiscriminate OR “parent-child” OR “parent child” OR “mother-child” OR “mother child” OR relationship OR bond.

 

Finally, Google Scholar searches were conducted using the search terms (a) FASD + attachment and (b) “prenatal alcohol” + attachment, and the first 10 pages of results were screened. The additional search of Google Scholar was conducted because, even though this platform is not recommended as a principal search system [54], we have found the first few pages of results (when sorted by relevance) to be fruitful in the past. This is further demonstrated by the addition of one eligible study from Google Scholar searches in this review that was not identified in the database searches.

### 2.4. Data Collection Process and Data Items

Independent searches were carried out by the first and second authors, using the same review protocol. Results of the searches were uploaded into Covidence (https://www.covidence.org/), an AI-powered systematic review software for deduplication and screening [55]. The authors (using Covidence) removed the duplicates, independently screened the titles, abstracts and full-text outputs from their searches, and the results were then compared and reconciled. Conflicts were resolved by discussion between the first and second authors, guided by the eligibility criteria of the study. Data extraction was undertaken by the second author, as there were no disagreements that required a third reviewer to be consulted. A data extraction sheet was employed to extract the data from the included studies (Table 1). The data extracted included the author(s) names, publication year, study setting, the tools employed, and the design of each study. Co-authors reviewed the data extraction sheets independently and evaluated the quality of included studies. The reference sections of included articles were also screened by the first and second authors to identify any articles that met the inclusion criteria of this review.

#### Critical Appraisal of Individual Sources of Evidence

Critical appraisal (by the first and second author) of the identified studies was undertaken using the Quality of Observational Cohort and Cross-Sectional Studies tool (QOCCSS) [67]. This tool includes a 14-item checklist which permits reviewers to categorise the qualities of studies based on the amount of information provided by authors. The included studies were independently appraised by the first and second authors. Following this, a discussion session was held to reconcile the appraisal scores. The QOCCSS does not produce an overall quality score. Rather, the process of checking these items is designed to help reviewers reflect on the overall quality of each paper [67]. Nevertheless, a total score was produced for this review based on the number of criteria met versus not met (shown in Table S1). Any items deemed not applicable were removed from this analysis, and an overall score was calculated with yeses as the numerator and the sum of yeses, noes, and not reported as the denominator, presented as a decimal.

### 2.5. Synthesis Approach

A narrative synthesis approach was employed to synthesise the findings from identified studies, as this approach provides an opportunity to qualitatively describe the findings [68,69]. The primary outcome of interest in each study was whether any of their data analyses had demonstrated a significant relationship between PAE and attachment styles. For example, if their cross-sectional analysis had demonstrated a significant correlation between the level of PAE and a continuous measure of attachment security, or if their case-control analysis had found that people with an FASD diagnosis were more likely to have a different attachment style than typically developing controls.

## 3. Results

### 3.1. Study Selection

**Figure 1:**
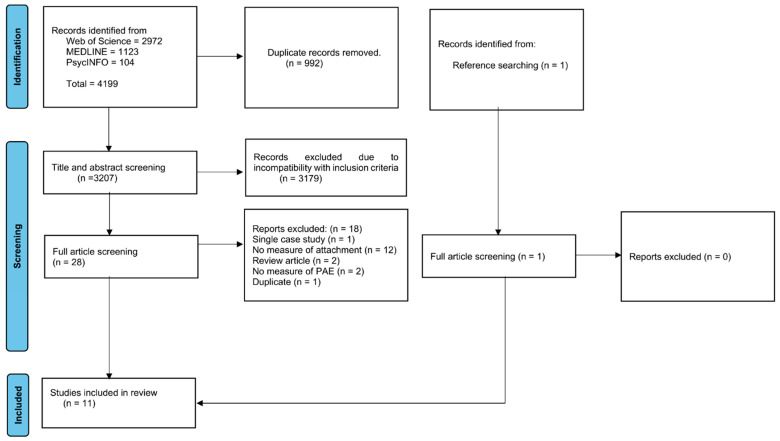
PRISMA flow diagram for systematic search.

The systematic search of databases yielded 4199 records. Covidence identified and removed 992 duplicates. The titles and abstracts of 3207 studies were screened for relevance, and 3179 were removed as they did not fit the inclusion criteria of this review. Meanwhile, manual searching using Google Scholar identified one additional article. Full article screening was conducted for 29 studies, and 11 met the criteria and were included in the final review. Of the 18 studies that were excluded in the final round, most (12) were excluded because they did not include a measure of attachment. The others were excluded because they did not include a measure of PAE or FASD (2), were review articles and therefore contained no new data (2), were single case studies (1), or were a duplicate of a study we had already included (1) (see Figure 1).

In total, 11 studies were included in the final review (see Table 1). Eight of these were peer-reviewed journal articles [56,58,59,60,61,63,64,65], and three were university dissertations from master’s [66] or doctoral [57,62] projects. Eight were conducted in the United States, one in Australia [64], one in South Africa [66], and one in Poland [65]. They were published between 1987 and 2021. The studies included a total of 1198 research participants: 324 individuals with PAE/FASD, 817 caregivers, and 57 typically developing control participants. Six of the studies were based on mother–child dyads [56,57,58,59,60,63], and in four of those studies, the toddlers were aged between 12 and 18 months [56,58,59,63]. In the other two dyad studies, the children were aged between four and six years [57,60]. One study was based on mothers only [64], one on children with PAE/FASD only [61], and three on adolescents or young adults only [62,65,66].

### 3.2. Quality of Studies

Based on the quality assessment tool used in this review (QOCCSS; [67]), the studies had a variable level of quality. The tool provides its score based on the study design and the details of reporting. The QOCCSS includes 14 items, and we calculated an overall score based on the items included versus the total number of applicable items in each study, which produced an overall score expressed as a decimal. The lowest score was 0.43 [64], and the highest score was 1 [58]. The mean score for all eight articles was 0.73, and the standard deviation was 0.17. Details are provided in Table S1. The studies tended to provide sufficient detail on exposures, measurements, and participant characteristics, but tended to overlook important confounding variables such as other pre- and post-natal exposures and adversities.

In all studies, attachment was measured with validated tests, most often observation-based methods such as the strange situation procedure or Attachment Q-Set. Some studies measured attachment from the caregiver’s perspective rather than the child’s perspective, so caregiver–infant attachment rather than infant/child–caregiver attachment (for more details on measures, see Section 3.2.2). However, none of the studies used a combination of observational and self-report tools, which has been recommended [70]. The FASD-related variable was sometimes a diagnosis on the fetal alcohol spectrum and was sometimes a measure of PAE. PAE was measured by maternal self-report in all studies that explored PAE rather than an official diagnosis. One study [63] used a combination of maternal report and toxicology screening.

#### 3.2.1. Measures of PAE and FASD

The studies differed in terms of how they operationalised and measured PAE or FASD. [56] used a self-report questionnaire that their participants (mothers of 12-month-old infants) completed about the frequency and quantity of their consumption of alcohol, caffeine, tobacco, and drugs before, during, and after pregnancy. Based on their responses, absolute alcohol intake in ounces per day was calculated for pre-pregnancy, during pregnancy, and after pregnancy. Wilson [57] conducted a follow-up of this study using the same cohort of mother–child dyads four years later, and they did not measure PAE again at this stage. O’Connor et al. [58] used a maternal self-report questionnaire at one-year post-partum. This was used to calculate the absolute alcohol score based on ounces per day and the average number of drinks per drinking occasion. Swanson et al. [59] used maternal self-reports for alcohol and drug use during pregnancy but provided little detail on how this information was obtained. PAE was operationalised as a binary variable (present or absent). O’Connor et al. [60] interviewed their participants and calculated their maximum drinks per drinking occasion, which was the measure of PAE in this study, using the procedure developed by [71]. Farina et al. [61] compared children with a diagnosis of what they called “an Alcohol Related Neurodevelopmental Disorder” against children without such a diagnosis. This was based on the caregiver report, and there is no mention of how these diagnoses were made, or which diagnostic criteria were used. Yumoto [62] screened for alcohol consumption during the prenatal stage using a timeline follow-back interview, and PAE was measured in absolute alcohol consumption per day. Bergin and McCollough [63] measured PAE during the prenatal stage and at delivery using urine toxicology screening and by maternal self-report during pregnancy. Rossen et al. [64] collected information about PAE at each trimester of pregnancy and eight weeks postpartum, but no information is provided about how this data was collected. Kornaszewska-Polak et al. [65] used the diagnosis of Fetal Alcohol Syndrome (FAS) as a binary variable to compare the cases with controls. FAS was diagnosed using the four-digit code system [72], and the control group was described simply as ‘excluded FAS’. There was no detail provided on how the control group was recruited or whether they had any PAE or other FASD diagnoses, nor whether the two groups differed in terms of their adoption or fostering histories. Kemp [66] compared dysmorphic with non-dysmorphic presentations of FASD. Dysmorphology was assessed by specialists using the Institute of Medicine criteria [73].

#### 3.2.2. Measures of Attachment

O’Connor et al. [56] used the SSP ([26]) to determine the attachment relationship of each of their mother–infant dyads. Wilson [57] was a follow-up of this cohort four years later, and at this stage utilised the Separation Anxiety Test (SAT to assess mother–child attachment. The SAT is a procedure that involves children describing their emotional reaction to illustrations depicting separations of children and parents of varying lengths [74]. O’Connor et al. [58] also used the SSP and the Mother–Child Ratings Scale [75] as a measure of child negative affect. Swanson et al. [59] and Kemp [66] measured attachment using the SSP. O’Connor et al. [60] used the Family Interaction Puzzle Task to measure mother supportive presence, child negative affect, and child coping, and the Attachment Q-Set [76], an observational measure similar to the SSP, to measure parent–child attachment. Bergin and McCollough [63] also used the Attachment Q-Set. Farina et al. [61] used the Attachment Security Questionnaire [77] as a measure of attachment, which is conceptually based on the Attachment Q-Set but uses caregiver report rather than observation. Yumoto [62] measured attachment in their adolescent participants using the Child Attachment Interview (CAI; [78]). The CAI is an observed interview with questions about a child’s relationship to their caregivers. Coding and attachment classification are based on non-verbal cues, as well as the child’s answers. Rossen et al. [64] measured attachment using caregiver report questionnaires during and after pregnancy: the Maternal Antenatal Attachment Scale (MAAS; [79]) and the Maternal Postnatal Attachment Scale (MPAS; [80]). Kornaszewska-Polak et al. [65] measured attachment using the Polish version of the Experience in Close Relationships Questionnaire—Revised [81], which provides a score for four dimensions of attachment: secure, preoccupied, dismissing–avoidant, and fearful–avoidant.

#### 3.2.3. The Relationship Between PAE/FASD and Attachment Security

As per the inclusion criteria, all 11 studies assessed the relationship between PAE/FASD and attachment. Most of the analyses used a measure of attachment security as the dependent variable (e.g., secure attachment as opposed to insecure or disorganised). Five of the 11 studies in this review found a significant relationship between PAE/FASD and attachment security in some way. O’Connor et al. [56] found that children with higher levels of PAE were more likely to display insecure attachment than those with lower levels. Kornaszewska-Polak et al. [65] found that young adults with a diagnosis of foetal alcohol syndrome were more likely to have an insecure attachment style than typically developing controls. Yumoto [62] found that the severity of maternal drinking problems during pregnancy was a predictor of disorganised attachment in adolescents with PAE. O’Connor et al. [58] and O’Connor et al. [60] found that PAE and attachment were not significantly related when no other variables were included in the model, but when caregiver and child factors were included as mediating variables, there was a significant model.

#### 3.2.4. Mediating Variables

The studies in this review differ in terms of their contribution to the aim of this review, which was to identify any published data on the relationship between PAE/FASD and infant/child–caregiver attachment. Some were not designed to focus on the relationship between PAE/FASD and attachment. For example, Swanson et al. [59] assessed the wider impact of prenatal substance exposure on attachment and related metrics. The study only met criteria for this review due to one, seemingly incidental, finding that PAE was not associated with attachment classification. Bergin et al. [63] and Rossen et al. [64] also studied the wider category of prenatal substance exposure, with some data presented on alcohol, while O’Connor et al. [56], Farina et al. [61], and Kornaszewska-Polak et al. [65] focused on PAE/FASD. However, two of the studies, O’Connor et al. [58] and O’Connor et al. [60], provide probably the most important contributions to our findings. These studies were designed to assess the relationship between PAE and attachment security in infants aged 12 months [58] and children aged 4–5 years [60], but they are also the only studies in this review to assess the impact of potential mediating variables. Following an earlier study by the same group [56], which was the first to address the relationship between PAE and attachment, the O’Connor team hypothesised that infant affect, postnatal alcohol consumption, and infant–mother interaction might mediate the relationship between PAE and attachment. In each of their two later studies, they used structural equation modelling to show that a higher level of PAE was related to negative infant affect, which was related to less-optimal mother–child interaction, which was then more likely to relate to an insecure attachment relationship in both 12 month old infants [58] and 4–5 year old children [60]. Although there is mixed evidence of a direct relationship between PAE and attachment security, it appears that children with PAE/FASD are at increased risk of insecure attachment, depending on their individual presentation and/or mediating factors. To put it another way, establishing a secure attachment relationship may be more challenging for the caregivers of children with PAE/FASD who exhibit high levels of irritability or negative affect.

#### 3.2.5. Other Variables

The studies in this review assessed the relationship between attachment and several other predictor variables aside from PAE/FASD. These are important to note here as potential confounders, although most of the variables were not significantly related to attachment. Swanson et al. [59] found no difference in attachment between children of biological parents and foster/kinship caregivers, but that caregiver intrusiveness (overstimulation, overinvolvement) was related to insecure attachment. O’Connor et al. [60] found a greater proportion of insecurely attached children in their sample of “high risk” (low-income, mostly single) mothers than in their previous (1987, 1992) samples of middle-class (mostly married) mothers. O’Connor and colleagues [58,60] also found no relationship between the following variables and attachment security: prenatal tobacco or caffeine; child’s gender or ethnicity; mothers’ IQ, education, age, work status, income, or socioeconomic status; father’s education or social class; number of siblings in the home; obstetrical complications; time spent nursing; postnatal alcohol consumption; and current alcohol consumption. Bergin and McCollough [63] found that quality of caregiving predicted attachment, whilst amounts of prenatal alcohol and cocaine exposure did not. Rossen et al. [64] found a relationship between maternal bonding with her baby during the prenatal stage and during the postnatal stage. Kornaszewska-Polak et al. [65] found that young adults diagnosed with foetal alcohol syndrome had higher scores for attachment avoidance than typically developing controls, but scores for attachment anxiety were not significantly different between those groups.

## 4. Discussion

The purpose of this scoping review was to identify all primary studies on the relationship between PAE/FASD and caregiver–infant attachment. Only 11 studies were identified that fit our inclusion criteria, and the studies yielded mixed results. Five of the studies showed that children or young people with a history of PAE/FASD were more likely to have an insecure or disorganised attachment compared to those without PAE/FASD. The other six studies found no significant relationship between PAE/FASD. These differences are due to unaccounted confounding factors in these studies and/or weak levels of interaction between the measured variables. For example, emotional regulation impacts attachment relationships significantly [82], and children with FASD are likely to have difficulties with emotional regulation. Within the five studies that found a significant relationship, the findings of two of these studies were only significant via a path that included infant irritability and caregiver–child interaction.

For many years, the attachment relationship has been described as crucial to parent–child interaction and the developing relationship [25,26]. These attachment behaviours develop from birth through various stages. It has been shown that, when the development of healthy attachment behaviour is impaired, it can have a significant impact on an individual’s behaviour, with long-term mental health consequences [83,84]. What was less well-understood was the nature of the attachment relationship with regard to parent–child dynamics when there is a pre-existing developmental challenge.

Some of the studies in this review found that children who had PAE and FASD were more likely to have a disorganised attachment style e.g., [56], yet other studies did not find this e.g., [59]. Studying other neurodevelopmental disorders as a guide to see how those with pre-existing deficits in neurological pathways (particularly affecting social communication and social relationships) are affected, the literature suggests that these individuals have a higher likelihood of an insecure attachment style [85]. In a study that compared a group with social communication deficits with controls [86], this was found not to be related to parenting. These results indicate that, where social communication pathways are damaged, attachment behaviour may also be inherently disturbed. This is particularly important when parenting is under scrutiny, especially in the case of care-experienced children, a group where studies [12,87] have demonstrated that children with FASD are overrepresented.

Evidence from FASD research and parental reports suggests that understanding attachment behaviours is key to the parent–child dynamic. Improvements in attachment are also possible through strategies that support the child’s management [88,89]. These improvements may not, however, be through traditional approaches to parenting. Children with PAE/FASD may benefit from alternative approaches to parenting and support, as their neurological deficits may limit the efficacy of traditional approaches [90]. For example, conditioning tasks have been shown not to work for individuals with FASD [91]; yet this is the approach advocated in many generic parent training approaches (e.g., Incredible Years; [92]). This explains why some parts of traditional parenting approaches may prove challenging in this population [90,93]. The parent may also present the child with inconsistent and disturbing approaches, leading to fear and apprehension towards the parent, even if that is not the intention. Understanding the individual primarily through the lens of their neurodevelopmental disorder helps to prevent inconsistent responses from caregivers. A relevant example from autism research is the concept of ‘double empathy’, which emphasises understanding the individual’s perspective rather than relying on traditional approaches [94,95]. Another factor, which may explain the variability in the observed findings of the few studies (included in this review), is the variability in the presentation of FASD. While individuals with dysmorphic features and significant cognitive deficits are more likely to exhibit severe behavioural and cognitive impairments, the range of deficits is broader among those without dysmorphic features [96]. This will mean that different samples may not be immediately comparable. Stratification by severity of presentation of the FASD and associated comorbid conditions may be necessary, especially where social communication is affected. Examples of associated comorbid conditions in this regard include sensory processing disorders, executive function disorders, and oppositional defiance disorder [97,98]. To ascertain broader needs, rather than considering everybody to have the same level of deficit, a better sampling frame with stratification of individuals may be needed.

## 5. Practical Implications

The findings of this scoping review present some practical implications. First, the studies identified within this review suggest the potential influence of PAE/FASD on the development of disorganised or insecure attachment in impacted children. Whilst the current available data does not lead to any firm conclusions and more work is needed, this does highlight the potential advantage of screening for PAE/FASD in children at risk of attachment disruption, such as looked-after children. Conversely, it may be useful to assess for attachment disorders in children impacted by PAE/FASD. This may help to improve service provision and lead to improved life outcomes for impacted children. Second, the findings from this review highlight the need for clinicians and other professionals to work with children using an approach that is neurodevelopmentally sensitive. This is important because the impact of PAE/FASD results in organic brain damage, which can then impact the behavioural outcomes seen in impacted children. Rather than attributing the behavioural presentation/attachment style to other external factors, findings from our review could inform practitioners of the advantage of querying prenatal brain damage. Furthermore, this review also indicates the necessity for the development and use of attachment assessment tools that capture possible mediating factors that may influence attachment styles, attachment security, and attachment disorders. As seen within several studies in this review, many of the methods employed were limited in scope and did not account for other confounding factors that may influence attachment security.

## 6. Limitations and Future Directions

This review was not pre-registered. Pre-registration can help demonstrate that the aims and hypotheses of a study are established prior to data collection and are not influenced by the findings. However, as this review was exploratory in nature and did not include any a priori hypotheses, the applicability of traditional pre-registration frameworks was limited. Nevertheless, best practice is to pre-register, and we did not do this. Another limitation is the narrow focus of this review. We explored whether PAE could lead directly to attachment disruption and considered whether the higher rate of attachment disruption in this population could be better explained by other factors, such as postnatal adversity. As mentioned above, this remains an open question. However, there are related topics that we chose not to focus on here, such as interventions and mechanisms of action, like parent–child relationships or emotional self-regulation. There is much work to be conducted on the topic of attachment in the FASD population that is beyond the scope of this review.

The outcome of this review is that firm conclusions are yet to be drawn on the question of whether PAE can impact attachment relationships. The literature to date has been primarily conducted in smaller groups with mixed methodology, and all studies have some limitations. Additionally, the samples within the identified studies in this review were mostly of Western origin and other populations, e.g., indigenous populations (where FASD is overrepresented), remain understudied. The studies included encompass a wide time scale in which they were conducted, with most published over a decade ago, and the complexities of parent–child relationships were not captured by the study designs and tools employed. The understanding of the relationship between adverse childhood experiences, vulnerability, and neurological disorder has become better understood in this timescale [9,11,19,72,99]. Due to the high rates of ACEs and other forms of adversity in this population, as evidenced in this literature, there is a need for further research to investigate the combined impact of multiple forms of pre and postnatal adversity on attachment relationships. Furthermore, the studies varied with respect to their qualities; for example, statistical adjustments for confounding variables were not implemented in all of the studies, and there was a heavy reliance on self-reporting of alcohol exposure, which would have benefited from the additional use of biomarkers where possible [100].

Those with FASD are overrepresented in populations where attachment difficulties are common [12,87]. A significant reason for children to be taken into care (especially in the UK) is parental drug and alcohol abuse [101,102], leading to rates of up to 27% of a looked-after children’s population being described as having probable FASD [87]. It is therefore vital to understand the attachment needs of this group and how further complicating adverse experiences add to a traumatic and disturbed parent–child relationship. To not do so will lead to clinical complications, placement breakdowns, and an increased likelihood of mental health deficits [103,104]. It has also been demonstrated that, where good relationships have been formed, the strengths of the child are built upon, providing better understanding for parents that long-term positive outcomes are seen [8,105,106].

This review provides an overview of the literature available in the area of PAE and attachment and provides a foundation for future work in this area. Further work is needed with more structured research, with wider sampling frames and stratification of samples. Further, more robust prospective methodological approaches are needed for robust conclusions to be made. Due to the significant impact of attachment difficulties on mental health presentations in children, an understanding of the dynamic and relationship between pre-existing neurological deficits common in FASD needs to be combined with the environmental secondary exposures to better understand how they impact upon the developing attachment relationship between parent and child. Environmental exposures to be considered to include, but are not limited to, adverse school experiences, neglect, abuse, multiple home placements, and lack of adequate support.

## 7. Conclusions

There is a significant interplay between neurology and environment in the development of the attachment relationship between parents/carers and those affected by PAE. This scoping review demonstrates that the evidence base is sparse and does not take into account more recent developments in the understanding of the relationship between adverse childhood experiences and neurological disorders. Evidence to date has shown mixed presentations, highlighting an urgent need to explore this further in what is an extremely vulnerable group. FASD is a condition that often co-occurs with a history of postnatal adversity, leading to a dual impact. Those with FASD tend to have been exposed to more factors that would typically influence attachment. It is therefore vital to develop a stronger evidence base, both to understand the inter-relationships between prenatal alcohol exposure, postnatal adversity, and attachment, an understanding of which can then lead to appropriate interventions.

## Figures and Tables

**Table 1:** Study characteristics.

Reference Location	Sample	Design	Aims	Measures	Results
[56] California, USA	N = 46 mother–infant dyads. Infants aged 12 months, mothers aged 30+ Predominantly white (92%), highly educated (72% college or more education) and middle class (100%).	Cross-sectional	To assess relationships between amount of alcohol consumed by mothers before and during pregnancy and attachment styles in infants at 12 months	Alcohol consumption in pregnancy was measured by self-report questionnaire at 12 months postpartum. Attachment was determined using the SSP—infants were categorised as either securely attached, insecure–resistant, insecure–avoidant, or disorganised.	Higher PAE scores in insecure attachment group.
[57] California, USA	41 mother (30+ years) child (5–6 years) dyads from the same sample as [56]. Mothers were 88% white, 64% college educated, 100% middle class, and their average IQ score was 117.	Follow-up, cross-sectional.	To assess the consistency of attachment classifications measured at 12 months using the Strange Situation Procedure and at 5–6 years using the Separation Anxiety Test (SAT). To measure the relationship between prenatal alcohol exposure and attachment style in children aged 5–6 using the SAT.	Attachment style was measured with the Separation Anxiety Test. PAE was measured by maternal self-report.	SSP at 1 year and SAT at 6 years did not result in the same attachment categorisations. No relationship between SAT classifications and PAE.
[58] California, USA	N = 44 firstborn infants (aged 1 year) of middle-class (100%) mothers aged 30+. English-speaking (first language), highly educated participants, with 70% who have achieved college or postgraduate education	Cross-sectional	To investigate possible mechanisms between PAE and insecure/disorganised attachment using structural equation modelling, including infant irritability, postnatal alcohol consumption, and mother-infant interaction. Attachment category was the dependant variable.	Alcohol consumption during and after pregnancy was measured by self-report questionnaire and operationalised as average drinks per drinking occasion. Child and maternal behaviours were rated using the Mother–Child Rating Scales. Attachment style was determined using the SSP. Attachment was dichotomous (secure or not), and alcohol was measured as average drinks per drinking occasion. Smoking and caffeine use during pregnancy were also measured.	Best-fitting model identified through structural equation modelling: PAE, negative infant affect, maternal responsiveness, less secure attachment.
[59] California, USA	N = 51 mother–toddler (aged 18 months) dyads. Participants’ mean age was 27.80 (range: 19–45), with time spent in education (mean: 11.24; range: 7–14). 39.2% were employed, and 82.4% were from minoritised ethnic groups.	Cross-sectional	To examine the relationships between prenatal substance exposure, current substance use, caregiver sensitivity and hostility, and attachment styles.	Substance use was measured using self-report. Attachment was determined using the SSP.	PAE by maternal report was not associated with attachment classification.
[60] California, USA	42 children (aged 4–5 years) of low-income ‘high-risk’ mothers. Mothers were English-speaking with age range of 21–44 years. Majority were high school graduates (38%), followed by those who have had some college education (31%). Five percent were college graduates, and 26% were non-high school graduates. Ethnicity-wise, 83% were African Americans, followed by seven percent Hispanics and 10% from ‘other’ ethnicities.	Cross-sectional	Used structural equation modelling to test the hypothesis that higher PAE would be related to insecure/disorganised attachment styles via infant negative affect and less-optimal mother–child interaction.	PAE was measured by interview and operationalised as maximum number of drinks per drinking session. Attachment style was measured using the Attachment Q-Set. Mother supportive presence, child negative affect, and child coping were measured using the Family Interaction Puzzle Task.	PAE predicted negative child affect, insecure attachment and lower levels of maternal emotional support of the child. When mothers of PAE children provided high levels of support, children had better coping skills and more secure attachment.
[61] Illinois, USA	29 Russian children (age 1–7) adopted by US families. 10 of the children were diagnosed with “an ARND”. 21% of the mothers completed high school (12th grade), followed by 14% who undertook one or two years of undergraduate studies; 21% had four years of undergraduate education, and 43% completed graduate education (one to three years).	Cross-sectional	To assess relationships between parenting stress, attachment security, child behavioural difficulties, length of institutionalisation, and ARNDs.	Attachment was measured using the caregiver-report Attachment Security Questionnaire. The children either had a diagnosis of ARND or they did not. But it is unclear how/when/where they were assessed, which criteria were used, or whether any of the non-diagnosed children had PAE.	Parenting stress was significantly correlated with insecure attachment and increased child behavioural difficulties. Attachment insecurity was significantly correlated with the severity of behaviour problems. No differences were found with regard to gender or diagnosis of ARND.
[62] Michigan, USA	N = 130 inner-city, African American adolescents (mean age = 14.3 years). Mothers were Low SES, 75% were unmarried, and their average number of years of education was 12.6.	Cross-sectional follow-up. Mothers had been recruited during pregnancy.	To assess the relationships between PAE, childhood trauma, attachment, and behavioural functioning in adolescents.	PAE measured by maternal interview during pregnancy. Attachment measured in adolescents using the Child Attachment Interview.	PAE was not related to attachment classification, but severity of maternal drinking problems during pregnancy did predict disorganised attachment.
[63] Ohio, USA	Low-income (“high risk”) mothers (n = 41) with their substance-exposed 12-month-olds. Majority of participants with substance exposed toddlers received Medicaid due to poverty (93%) and were of African American origin (86%).	Matched pairs	Substance-exposed infants were compared with a nonexposed group matched for other risk factors (poverty, race, gender, gestational age, birth weight, mothers’ age at delivery, and parity). Outcomes measured were attachment, maternal sensitivity, quality of caregiving.	PAE was measured with urine toxicology at antenatal care and postnatally, as well as by interview. Maternal sensitivity and involvement were analysed from 2 h of videotaped interaction. Attachment was assessed using the Attachment Q-Set.	Attachment security and quality of caregiving were quite low for both groups, with no significant differences. Regression analyses revealed that quality of caregiving predicted attachment, but amount of alcohol and cocaine exposure did not.
[64] Rossen 2016 New South Wales and Western Australia, Australia	372 pregnant women (data collected throughout pregnancy and at 8 weeks postpartum) Maternal age (median) was 33.0 years; 1.1% had indigenous background and 2.5% were indigenous, and 41.1% were born overseas.	Cross-sectional	To assess the relationships between pre- and post-natal bonding between mothers and infants, and the impact of substance use on bonding.	Mothers’ feelings of attachment towards their infant were measured using the Maternal Antenatal Attachment Scale, and Maternal Postnatal Attachment Scale. Depression and anxiety were measured using the Edinburgh Antenatal and Postnatal Depression Scale and the Depression and Anxiety Scales. Information about alcohol and drug use was collected from mothers at each trimester and eight weeks postpartum.	Higher antenatal bonding predicted higher postnatal bonding. Maternal depressive symptoms in pregnancy predicted better mother-infant bonding 8 weeks. No evidence that alcohol use during pregnancy is associated with postpartum bonding (alcohol tended to be low level).
[65] Silesian Voivodeship, Poland	Young adults with foetal alcohol syndrome (n = 30) and control group without FAS (n = 30, although in places n = 39) Participants with confirmed FASD	Case-control with longitudinal follow-up	Longitudinal design.	FAS in children was diagnosed using the 4-digit code system between 1990 and 2000. Attachment style in young adults was assessed with the Polish version of the Experience in Close Relationships—Revised questionnaire in 2017.	Adults diagnosed with FAS achieved higher scores in insecure attachment than adults without FAS.
[66] Western Cape, South Africa	N = 77 adolescents in total: 50 with FASD, 27 controls with minimal or no PAE.	Cross-sectional follow-up study	To examine the association between FASD facial dysmorphism and insecure, as well as disorganised, attachment. To examine the extent to which FASD facial dysmorphism was associated with emotion regulation difficulties in adolescence after controlling for the effects of attachment security in infancy.	Attachment measured using the Strange Situation Procedure. FASD dysmorphism assessed clinically by specialist dysmorphologists using the Institute of Medicine Criteria.	No difference in attachment security between dysmorphic and non-dysmorphic groups. Did not compare FASD and control groups on attachment security using a statistical test, but the control group did have the highest rate of secure attachment.

PAE: prenatal alcohol exposure; SSP: strange situation procedure; FASD: Foetal Alcohol Spectrum Disorder; ARND: Alcohol-Related Neurodevelopmental Disorder; FAS: Foetal Alcohol Syndrome; SAT: Separation Anxiety Test; USA: United States of America.

## Supplementary Materials

The following supporting information can be downloaded at: https://www.mdpi.com/article/10.3390/children12091133/s1, Table S1.

## References

[1] Goodlett C.R., Horn K.H., Zhou F.C. (2005). Alcohol teratogenesis: Mechanisms of damage and strategies for intervention. Exp. Biol. Med..

[2] Lipinski R.J., Hammond P., O’Leary-Moore S.K., Ament J.J., Pecevich S.J., Jiang Y., Budin F., Parnell S.E., Suttie M., Godin E.A. (2012). Ethanol-induced face-brain dysmorphology patterns are correlative and exposure-stage dependent. PLoS ONE.

[3] Lange S., Probst C., Gmel G., Rehm J., Burd L., Popova S. (2017). Global prevalence of fetal alcohol spectrum disorder among children and youth: A systematic review and meta-analysis. JAMA Pediatr..

[4] Cook J.L., Green C.R., Lilley C.M., Anderson S.M., Baldwin M.E., Chudley A.E., Conry J.L., LeBlanc N., Loock C.A., Lutke J. (2016). Fetal alcohol spectrum disorder: A guideline for diagnosis across the lifespan. Can. Med. Assoc. J..

[5] Goodlett C.R., Horn K.H. (2001). Mechanisms of alcohol-induced damage to the developing nervous system. Alcohol Res. Health.

[6] Ungerer M., Knezovich J., Ramsay M. (2013). In utero alcohol exposure, epigenetic changes, and their consequences. Alcohol Res. Curr. Rev..

[7] McQuire C., Daniel R., Hurt L., Kemp A., Paranjothy S. (2020). The causal web of foetal alcohol spectrum disorders: A review and causal diagram. Eur. Child Adolesc. Psychiatry.

[8] Flannigan K., Wrath A., Ritter C., McLachlan K., Harding K.D., Campbell A., Reid D., Pei J. (2021). Balancing the story of fetal alcohol spectrum disorder: A narrative review of the literature on strengths. Alcohol. Clin. Exp. Res..

[9] Price A., Cook P.A., Norgate S., Mukherjee R. (2017). Prenatal alcohol exposure and traumatic childhood experiences: A systematic review. Neurosci. Biobehav. Rev..

[10] Tan G.K.Y., Symons M., Fitzpatrick J., Connor S.G., Cross D., Pestell C.F. (2022). Adverse childhood experiences, associated stressors and comorbidities in children and youth with fetal alcohol spectrum disorder across the justice and child protection settings in Western Australia. BMC Pediatr..

[11] Rockhold M.N., Handley E.D., Petrenko C.L.M. (2024). Understanding the intersection of prenatal alcohol exposure and postnatal adversity: A systematic review from a developmental psychopathology lens. Alcohol Clin. Exp. Res..

[12] Popova S., Lange S., Shield K., Burd L., Rehm J. (2019). Prevalence of fetal alcohol spectrum disorder among special subpopulations: A systematic review and meta-analysis. Addiction.

[13] Del Corral Winder S., Rinner A., Dickson A. (2023). FASD and Young Children in Foster Care. Child Welf..

[14] Pelech W., Badry D., Daoust G. (2013). It takes a team: Improving placement stability among children and youth with Fetal Alcohol Spectrum Disorder in care in Canada. Child. Youth Serv. Rev..

[15] Lange S., Shield K., Rehm J., Popova S. (2013). Prevalence of fetal alcohol spectrum disorders in child care settings: A meta-analysis. Pediatrics.

[16] Bower C., Watkins R.E., Mutch R.C., Marriott R., Freeman J., Kippin N.R., Safe B., Pestell C., Cheung C.S.C., Shield H. (2018). Fetal alcohol spectrum disorder and youth justice: A prevalence study among young people sentenced to detention in Western Australia. BMJ Open.

[17] Mela M., Wall L., Buttinger P., DesRoches A., Wrath A.J. (2022). Rates and implications of fetal alcohol spectrum disorder among released offenders with mental disorder in Canada. Behav. Sci. Law.

[18] Brown N.N., Gudjonsson G., Connor P. (2011). Suggestibility and Fetal Alcohol Spectrum Disorders: I’ll tell you anything you want to hear. J. Psychiatry Law.

[19] Gilbert D.J., Allely C.S., Gudjonsson G., Mukherjee R.A.S., Cook P.A. (2024). Immediate and repeat interrogative suggestibility in a sample of adolescents with fetal alcohol spectrum disorder. Divers. Incl. Res..

[20] Gilbert D.J., Allely C., Mukherjee R., Gudjonsson G., Novick Brown N., Brown J., McGinn V., Cook P. (2025). An Empirical Examination of Confabulation in Adolescents with Fetal Alcohol Spectrum Disorder (FASD). J. Pediatr. Neuropsychol..

[21] Leszko M., Keenan-Devlin L., Adam E.K., Buss C., Grobman W., Simhan H., Wadhwa P., Mroczek D.K., Borders A. (2020). Are personality traits associated with smoking and alcohol use prior to and during pregnancy?. PLoS ONE.

[22] Ordenewitz L.K., Weinmann T., Schlüter J.A., Moder J.E., Jung J., Kerber K., Greif-Kohistani N., Heinen F., Landgraf M.N. (2021). Evidence-based interventions for children and adolescents with fetal alcohol spectrum disorders–a systematic review. Eur. J. Paediatr. Neurol..

[23] Goldberg S., Muir R., Kerry J. (1995). Attachment theory. Social, Developmental and Clinical Perspectives.

[24] Keller H. (2018). Universality claim of attachment theory: Children’s socioemotional development across cultures. Proc. Natl. Acad. Sci. USA.

[25] Bowlby J. (1969). Attachment and Loss.

[26] Ainsworth M.D.S., Blehar M.C., Waters E., Wall S.N. (1978). Patterns of Attachment: A Psychological Study of the Strange Situation.

[27] Main M., Solomon J., Brazelton T.B., Yogman M.W. (1986). Discovery of an insecure-disorganized/disoriented attachment pattern. Affective Development in Infancy.

[28] Cyr C., Euser E.M., Bakermans-Kranenburg M.J., Van Ijzendoorn M.H. (2010). Attachment security and disorganization in maltreating and high-risk families: A series of meta-analyses. Dev. Psychopathol..

[29] Crowell J.A., Treboux D. (1995). A review of adult attachment measures: Implications for theory and research. Soc. Dev..

[30] Jewell T., Gardner T., Susi K., Watchorn K., Coopey E., Simic M., Fonagy P., Eisler I. (2019). Attachment measures in middle childhood and adolescence: A systematic review of measurement properties. Clin. Psychol. Rev..

[31] Carretero-Dios H., Pérez C. (2007). Standards for the development and review of instrumental studies: Considerations about test selection in psychological research. Int. J. Clin. Health Psychol..

[32] Acevedo-Polakovich I.D., Reynaga-Abiko G., Garriott P.O., Derefinko K.J., Wimsatt M.K., Gudonis L.C., Brown T.L. (2007). Beyond instrument selection: Cultural considerations in the psychological assessment of US Latinas/os. Prof. Psychol. Res. Pract..

[33] Waters E., Hamilton C.E., Weinfield N.S. (2000). The stability of attachment security from infancy to adolescence and early adulthood: General introduction. Child Dev..

[34] Fraley R.C., Roisman G.I. (2019). The development of adult attachment styles: Four lessons. Curr. Opin. Psychol..

[35] Ranson K.E., Urichuk L.J. (2008). The effect of parent–child attachment relationships on child biopsychosocial outcomes: A review. Early Child Dev. Care.

[36] Jethava V., Kadish J., Kakonge L., Wiseman-Hakes C. (2022). Early attachment and the development of social communication: A neuropsychological approach. Front. Psychiatry.

[37] Tryphonopoulos P.D., Letourneau N., Ditommaso E. (2014). Attachment and caregiver-infant interaction: A review of observational-assessment tools. Infant Ment. Health J..

[38] Delius A., Bovenschen I., Spangler G. (2008). The inner working model as a “theory of attachment”: Development during the preschool years. Attach. Hum. Dev..

[39] Biringen Z., Altenhofen S., Aberle J., Baker M., Brosal A., Bennett S., Coker E., Lee C., Meyer B., Moorlag A. (2012). Emotional availability, attachment, and intervention in center-based child care for infants and toddlers. Dev. Psychopathol..

[40] Potter-Dickey A., Letourneau N., de Koning A.P.J. (2020). Associations between neurodevelopmental disorders and attachment patterns in preschool-aged children: Systematic review. Curr. Dev. Disord. Rep..

[41] Beckman L., Janson S., von Kobyletzki L. (2016). Associations between neurodevelopmental disorders and factors related to school, health, and social interaction in schoolchildren: Results from a Swedish population-based survey. Disabil. Health J..

[42] Eiden R.D., Edwards E.P., Leonard K.E. (2004). Predictors of effortful control among children of alcoholic and nonalcoholic fathers. J. Stud. Alcohol.

[43] Rockhold M.N., Kautz-Turnbull C., Handley E.D., Petrenko C.L.M. (2023). The trauma experiences of children with fetal alcohol spectrum disorders: Developmental outcomes utilizing a threat/deprivation child adversity framework. Alcohol Clin. Exp. Res..

[44] Kambeitz C., Klug M.G., Greenmyer J., Popova S., Burd L. (2019). Association of adverse childhood experiences and neurodevelopmental disorders in people with fetal alcohol spectrum disorders (FASD) and non-FASD controls. BMC Pediatr..

[45] Mattson J.T., Thorne J.C., Kover S.T. (2022). Parental interaction style, child engagement, and emerging executive function in fetal alcohol spectrum disorders (FASD). Child Neuropsychol..

[46] Lowe J., Handmaker N., Aragón C. (2006). Impact of mother interactive style on infant affect among babies exposed to alcohol in utero. Infant Ment. Health J..

[47] Dozier M., Lindhiem O., Lewis E., Bick J., Bernard K., Peloso E. (2009). Effects of a foster parent training program on young children’s attachment behaviors: Preliminary evidence from a randomized clinical trial. Child Adolesc. Soc. Work J..

[48] Lieberman A.F., Weston D.R., Pawl J.H. (1991). Preventive intervention and outcome with anxiously attached dyads. Child Dev..

[49] Kohlhoff J., Lieneman C., Cibralic S., Traynor N., McNeil C.B. (2022). Attachment-based parenting interventions and evidence of changes in toddler attachment patterns: An overview. Clin. Child Fam. Psychol. Rev..

[50] Spieker S.J., Oxford M.L., Kelly J.F., Nelson E.M., Fleming C.B. (2012). Promoting first relationships: Randomized trial of a relationship-based intervention for toddlers in child welfare. Child Maltreatment.

[51] Munn Z., Peters M.D.J., Stern C., Tufanaru C., McArthur A., Aromataris E. (2018). Systematic review or scoping review? Guidance for authors when choosing between a systematic or scoping review approach. BMC Med. Res. Methodol..

[52] Anderson S., Allen P., Peckham S., Goodwin N. (2008). Asking the right questions: Scoping studies in the commissioning of research on the organisation and delivery of health services. Health Res. Policy Syst..

[53] Tricco A.C., Lillie E., Zarin W., O’Brien K.K., Colquhoun H., Levac D., Moher D., Peters M.D.J., Horsley T., Weeks L. (2018). PRISMA extension for scoping reviews (PRISMA-ScR): Checklist and explanation. Ann. Intern. Med..

[54] Gusenbauer M., Haddaway N.R. (2020). Which academic search systems are suitable for systematic reviews or meta-analyses? Evaluating retrieval qualities of Google Scholar, PubMed, and 26 other resources. Res. Synth. Methods.

[55] Babineau J. (2014). Product review: Covidence (systematic review software). J. Can. Health Libr. Assoc..

[56] O’Connor M.J., Sigman M., Brill N. (1987). Disorganization of attachment in relation to maternal alcohol consumption. J. Consult. Clin. Psychol..

[57] Wilson J. (1990). Stability of Attachment Classification from One to Six Years of Age in a Sample at Risk for Disorganized Attachment.

[58] O’Connor M.J., Sigman M., Kasari C. (1992). Attachment behavior of infants exposed prenatally to alcohol: Mediating effects of infant affect and mother-infant interaction. Dev. Psychopathol..

[59] Swanson K., Beckwith L., Howard J. (2000). Intrusive caregiving and quality of attachment in prenatally drug-exposed toddlers and their primary caregivers. Attach. Hum. Dev..

[60] O’Connor M.J., Kogan N., Findlay R. (2002). Prenatal alcohol exposure and attachment behavior in children. Alcohol. Clin. Exp. Res..

[61] Farina L., Leifer M., Chasnoff I.J. (2004). Attachment and behavioural difficulties in internationally adopted Russian children. Adopt. Foster..

[62] Yumoto C. (2008). Attachment representation in inner-city African American adolescents. Diss. Abstr. Int. Sect. B Sci. Eng..

[63] Bergin C., McCollough P. (2009). Attachment in substance-exposed toddlers: The role of caregiving and exposure. Infant Ment. Health J. Off. Publ. World Assoc. Infant Ment. Health.

[64] Rossen L., Hutchinson D., Wilson J., Burns L., Olsson C.A., Allsop S., Elliott E.J., Jacobs S., Macdonald J.A., Mattick R.P. (2016). Predictors of postnatal mother-infant bonding: The role of antenatal bonding, maternal substance use and mental health. Arch. Womens Ment. Health.

[65] Kornaszewska-Polak M., Klecka M., Janas-Kozik M., Palicka I. (2019). Quality of attachment in adults diagnosed with foetal alcohol syndrome. Psychiatr. Psychol. Klin. J. Psychiatry Clin. Psychol..

[66] Kemp A. (2021). Attachment and Emotion Regulation in Adolescents with FAS-Dysmorphism.

[67] NIH (2013). Quality Assessment Tool for Observational Cohort and Cross-Sectional Studies. https://www.nhlbi.nih.gov/health-topics/study-quality-assessment-tools.

[68] Lisy K., Porritt K. (2016). Narrative synthesis: Considerations and challenges. JBI Evid. Implement..

[69] Popay J., Roberts H., Sowden A., Petticrew M., Arai L., Rodgers M., Britten N., Roen K., Duffy S. (2006). Guidance on the conduct of narrative synthesis in systematic reviews. Prod. ESRC Methods Progr. Version.

[70] Trombetta T., Giordano M., Santoniccolo F., Vismara L., Della Vedova A.M., Rollè L. (2021). Pre-natal attachment and parent-to-infant attachment: A systematic review. Front. Psychol..

[71] Day N.L., Robles N. (1989). Methodological issues in the measurement of substance use. Ann. N. Y. Acad. Sci..

[72] Astley S.J. (2004). Diagnostic Guide for Fetal Alcohol Spectrum Disorders: The 4-Digit Diagnostic Code.

[73] Hoyme H.E., May P.A., Kalberg W.O., Kodituwakku P., Gossage J.P., Trujillo P.M., Buckley D.G., Miller J.H., Aragon A.S., Khaole N. (2005). A practical clinical approach to diagnosis of fetal alcohol spectrum disorders: Clarification of the 1996 institute of medicine criteria. Pediatrics.

[74] Klagsbrun M., Bowlby J. (1976). Responses to separation from parents: A clinical test for young children. Br. J. Proj. Psychol. Personal. Study.

[75] Crawley S.B., Spiker D. (1983). Mother-child interactions involving two-year-olds with Down syndrome: A look at individual differences. Child Dev..

[76] Waters E., Deane K.E. (1985). Defining and assessing individual differences in attachment relationships: Q-methodology and the organization of behavior in infancy and early childhood. Monogr. Soc. Res. Child Dev..

[77] Chisholm K. (1998). A three year follow-up of attachment and indiscriminate friendliness in children adopted from Romanian orphanages. Child Dev..

[78] Target M., Fonagy P., Shmueli-Goetz Y. (2003). Attachment representations in school-age children: The development of the Child Attachment Interview (CAI). J. Child Psychother..

[79] Condon J.T. (1993). The assessment of antenatal emotional attachment: Development of a questionnaire instrument. Br. J. Med. Psychol..

[80] Condon J.T., Corkindale C.J. (1998). The assessment of parent-to-infant attachment: Development of a self-report questionnaire instrument. J. Reprod. Infant Psychol..

[81] Lubiewska K., Głogowska K., Mickiewicz K., Wojtynkiewicz E., Izdebski P., Wiśniewski C. (2016). Skala Experience in Close Relationships-Revised: Struktura, rzetelność oraz skrócona wersja skali w polskiej próbie. Psychol. Rozw..

[82] Roque L., Veríssimo M., Fernandes M., Rebelo A. (2013). Emotion regulation and attachment: Relationships with children’s secure base, during different situational and social contexts in naturalistic settings. Infant Behav. Dev..

[83] Creeden K. (2009). How trauma and attachment can impact neurodevelopment: Informing our understanding and treatment of sexual behaviour problems. J. Sex. Aggress..

[84] Shaw S.K., Dallos R. (2005). Attachment and adolescent depression: The impact of early attachment experiences. Attach. Hum. Dev..

[85] Rutgers A.H., Bakermans-Kranenburg M.J., van Ijzendoorn M.H., van Berckelaer-Onnes I.A. (2004). Autism and attachment: A meta-analytic review. J. Child Psychol. Psychiatry.

[86] Rutgers A.H., Van Ijzendoorn M.H., Bakermans-Kranenburg M.J., Swinkels S.H.N., Van Daalen E., Dietz C., Naber F.B.A., Buitelaar J.K., Van Engeland H. (2007). Autism, attachment and parenting: A comparison of children with autism spectrum disorder, mental retardation, language disorder, and non-clinical children. J. Abnorm. Child Psychol..

[87] Gregory G., Reddy V., Young C. (2015). Identifying children who are at risk of FASD in Peterborough: Working in a community clinic without access to gold standard diagnosis. Adopt. Foster..

[88] Mukherjee R., Wray E., Commers M., Hollins S., Curfs L. (2013). The impact of raising a child with FASD upon carers: Findings from a mixed methodology study in the UK. Adopt. Foster..

[89] Mohamed Z., Carlisle A.C.S., Livesey A.C., Mukherjee R.A.S. (2020). Carer stress in Fetal Alcohol Spectrum Disorders: The implications of data from the UK national specialist FASD clinic for training carers. Adopt. Foster..

[90] Price A.D., Mukherjee R.A.S., Webster A., Tate D., Allely C.S., Brown S., Buckard J., Burd L., Butcher S., Shields J. (2023). Development and pre-feasibility testing of specific: A psychoeducation programme for caregivers of children with fetal alcohol spectrum disorder (FASD). J. Child Fam. Stud..

[91] Kodituwakku P., Kodituwakku E. (2014). Cognitive and behavioral profiles of children with fetal alcohol spectrum disorders. Curr. Dev. Disord. Rep..

[92] Pidano A.E., Allen A.R. (2015). The Incredible Years series: A review of the independent research base. J. Child Fam. Stud..

[93] Petrenko C.L.M., Pandolfino M.E., Roddenbery R. (2016). The Association Between Parental Attributions of Misbehavior and Parenting Practices in Caregivers Raising Children with Prenatal Alcohol Exposure: A Mixed-Methods Study. Res. Dev. Disabil..

[94] Cheang R.T.S., Skjevling M., Blakemore A.I.F., Kumari V., Puzzo I. (2024). Do you feel me? Autism, empathic accuracy and the double empathy problem. Autism.

[95] Mitchell P., Sheppard E., Cassidy S. (2021). Autism and the double empathy problem: Implications for development and mental health. Br. J. Dev. Psychol..

[96] Price A., Allely C., Mukherjee R. (2024). Fetal alcohol spectrum disorders: Where we have come from, trends, and future directions. Minerva Pediatr..

[97] Patel M., Agnihotri S., Hawkins C., Levin L., Goodman D., Simpson A. (2020). Identifying Fetal Alcohol Spectrum Disorder and psychiatric comorbidity for children and youth in care: A community approach to diagnosis and treatment. Child. Youth Serv. Rev..

[98] Popova S., Lange S., Shield K., Mihic A., Chudley A.E., Mukherjee R.A.S., Bekmuradov D., Rehm J. (2016). Comorbidity of fetal alcohol spectrum disorder: A systematic review and meta-analysis. Lancet.

[99] Minnis H. (2024). Abuse, neglect and neurodevelopment across the life course: What can paediatricians and child psychiatrists do about this together? The Illingworth-Rees keynote lecture 2023. Arch. Dis. Child..

[100] Corrales-Gutierrez I., Gomez-Baya D., Leon-Larios F., Medero-Canela R., Marchei E., Mendoza-Berjano R., García-Algar Ó. (2023). Alcohol consumption assessed by a biomarker and self-reported drinking in a sample of pregnant women in the south of Europe: A comparative study. Toxics.

[101] Gilbert D.J., Mukherjee R.A.S., Kassam N., Cook P.A. (2021). Exploring the experiences of social workers in working with children suspected to have fetal alcohol spectrum disorders. Adopt. Foster..

[102] HomeOffice (2003). Advisory Council on the Misuse of Drugs. Hidden Harm—Responding to the Needs of Children of Problem Drug Users. https://www.gov.uk/government/publications/amcd-inquiry-hidden-harm-report-on-children-of-drug-users.

[103] Petrenko C.L.M., Tahir N., Mahoney E.C., Chin N.P. (2014). Prevention of Secondary Conditions in Fetal Alcohol Spectrum Disorders: Identification of Systems-Level Barriers. Matern. Child Health J..

[104] Streissguth A.P., Bookstein F.L., Barr H.M., Sampson P.D., O’Malley K., Young J.K. (2004). Risk factors for adverse life outcomes in fetal alcohol syndrome and fetal alcohol effects. J. Dev. Behav. Pediatr..

[105] Kautz-Turnbull C., Adams T.R., Petrenko C.L.M. (2022). The strengths and positive influences of children with fetal alcohol spectrum disorders. Am. J. Intellect. Dev. Disabil..

[106] Kully-Martens K., McNeil A., Pei J., Rasmussen C. (2022). Toward a strengths-based cognitive profile of children with fetal alcohol spectrum disorders: Implications for intervention. Curr. Dev. Disord. Rep..

