# Supplementary material for: The Relationship Between Prenatal Alcohol Exposure and Infant/Child–Caregiver Attachment: A Scoping Review
Source: Children (Basel). 2025 Aug 27;12(9):1133. doi: 10.3390/children12091133 (PMC12468745; doi:10.3390/children12091133)
Supplement: Supplementary file 1 [file children-12-01133-s001.zip › children-3782440-supplementary.pdf]

S1: Supplementary table S1

Criteria	[56]	[57]	[58]	[59]	[60]	[61]	[62]	[63]	[64]	[65]	[66]
1. Was the research question or objective in this paper clearly stated?	Yes	Yes	Yes	Yes	Yes	Yes	Yes	Yes	Yes	Yes	Yes
2. Was the study population clearly specified and defined?	No	Yes	Yes	Yes	Yes	Yes	Yes	Yes	Yes	Yes	Yes
3. Was the participation rate of eligible persons at least 50%?	Yes	Yes	Yes	Yes	N/A	Yes	NR	NR	No	NR	Yes
4. Were all the subjects selected or recruited from the same or similar populations (including the same time period)? Were inclusion and exclusion criteria for being in the study prespecified and applied uniformly to all participants?	Yes	Yes	Yes	Yes	Yes	Yes	Yes	Yes	Yes	Yes	Yes
5. Was a sample size justification, power description, or variance and effect estimates provided?	No	No	Yes	No	No	Yes	No	No	No	No	Yes
6. For the analyses in this paper, were the exposure(s) of interest measured prior to the outcome(s) being measured?	Yes	Yes	Yes	Yes	Yes	Yes	Yes	Yes	Yes	Yes	Yes
7. Was the timeframe sufficient so that one could reasonably expect to see an association between exposure and outcome if it existed?	Yes	Yes	Yes	Yes	Yes	Yes	Yes	Yes	Yes	Yes	Yes
8. For exposures that can vary in amount or level, did the study examine different levels of the exposure as related to the outcome (e.g., categories of exposure, or exposure measured as continuous variable)?	Yes	Yes	Yes	Yes	Yes	No	Yes	Yes	No	No	Yes
9. Were the exposure measures (independent variables) clearly defined, valid, reliable, and implemented consistently across all study participants?	Yes	Yes	Yes	Yes	Yes	No	Yes	Yes	Yes	Yes	Yes
10. Was the exposure(s) assessed more than once over time?	N/A	No	N/A	No	No	No	No	Yes	No	No	N/A
11. Were the outcome measures (dependent variables) clearly defined, valid, reliable, and implemented consistently across all study participants?	Yes	Yes	Yes	Yes	Yes	No	Yes	Yes	No	Yes	Yes
12. Were the outcome assessors blinded to the exposure status of participants?	Yes	Yes	Yes	No	Yes	No	Yes	Yes	No	NR	Yes
13. Was loss to follow-up after baseline 20% or less?	N/A	Yes	N/A	N/A	N/A	N/A	No	Yes	No	N/A	No
14. Were key potential confounding variables measured and adjusted statistically for their impact on the relationship between exposure(s) and outcome(s)?	No	No	Yes	No	Yes	No	Yes	Yes	No	No	Yes
Total score	9/12=.75	11/14=.79	12/12=1	9/13=.69	10/12=.83	7/13=.54	10/14=.71	12/14=.86	6/14=.43	7/13=.54	12/13=.92

NR: Not Reported

N/A = Not applicable
